# Synthesis and Biological Evaluation of a New Acyclic Pyrimidine Derivative as a Probe for Imaging Herpes Simplex Virus Type 1 Thymidine Kinase Gene Expression

**DOI:** 10.3390/molecules18078535

**Published:** 2013-07-19

**Authors:** Andrijana Meščić, Thomas Betzel, Adrienne Müller, Roger Slavik, Stjepko Čermak, Silvana Raić-Malić, Simon M. Ametamey

**Affiliations:** 1 Department of Organic Chemistry, Faculty of Chemical Engineering and Technology, University of Zagreb, Marulićev trg 20, P.O. Box 177, HR-10 000 Zagreb, Croatia; E-Mails: amescic@fkit.hr; 2 Center for Radiopharmaceutical Sciences, ETH Zurich (Swiss Federal Institute of Technology), Wolfgang-Pauli-Strasse 10, CH-8093 Zurich, Switzerland; E-Mails: thomas.betzel@pharma.ethz.ch (T.B.); adrienne.mueller@pharma.ethz.ch (A.M.); roger.slavik@pharma.ethz.ch (R.S.); scermak@student.ethz.ch (S.Č.)

**Keywords:** acyclic pyrimidine nucleoside analogues, PCV-like side chain, fluorination, radiosynthesis, positron emission tomography (PET), HSV1-TK

## Abstract

With the idea of finding a more selective radiotracer for imaging herpes simplex virus type 1 thymidine kinase (HSV1-tk) gene expression by means of positron emission tomography (PET), a novel [^18^F]fluorine radiolabeled pyrimidine with 4-hydroxy-3-(hydroxymethyl)butyl side chain at *N*-1 (HHB-5-[^18^F]FEP) was prepared and evaluated as a potential PET probe. Unlabeled reference compound, HHB-5-FEP, was synthesized *via* a five-step reaction sequence starting from 5-(2-acetoxyethyl)-4-methoxypyrimidin-2-one. The radiosynthesis of HHB-[^18^F]-FEP was accomplished by nucleophilic radiofluorination of a tosylate precursor using [^18^F]fluoride-cryptate complex in 45% ± 4 (n = 4) radiochemical yields and high purity (>99%). The biological evaluation indicated the feasibility of using HHB-5-[^18^F]FEP as a PET radiotracer for monitoring HSV1-tk expression *in vivo*.

## 1. Introduction

**Figure 1:**
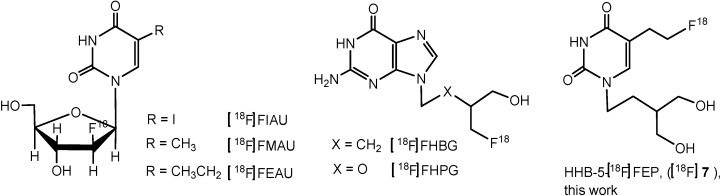
Structures of some pyrimidine and purine nucleoside analogues and HHB-5-[^18^F]FEP.

Positron emission tomography (PET) is a noninvasive imaging modality for the *in vivo* visualization of various metabolic processes such as cellular proliferation, HSV1-tk reporter gene expression [1,2,3], determination of receptor concentrations and the assessment of treatment response to therapy [4,5,6]. A paradigm for the non-invasive imaging of transgene expression involves the appropriate combination of a reporter gene and a reporter substrate or probe [7]. In essence, the reporter gene product selectively converts a reporter probe into a negatively charged metabolite that is trapped and accumulates within the transduced cells as it is unable to cross the cell membrane [3]. The accumulation of radioactivity within the transfected cell can be imaged by PET. The most studied reporter gene for the visualization of gene expression in animals and humans is herpes virus type 1 thymidine kinase (HSV1-tk) which is visualized by its enzyme product HSV1-TK. A number of ^18^F-labeled pyrimidine ([^18^F]-FIAU, [^18^F]-FMAU [8,9], [^18^F]-FEAU [10]) and purine ([^18^F]FHBG [11], [^18^F]FHPG [12]) based nucleosides have shown promise as reporter probes for non-invasive imaging of HSV1-tk gene expression (Figure 1).

In recent years, [^18^F]FEAU has emerged as a tracer with improved sensitivity and selectivity for imaging HSV1-tk expressing cells [13,14]. Whereas purine [15] and pyrimidine [16] nucleoside analogues have found application as imaging agents for imaging HSV1-tk expression, cellular proliferation is mainly imaged with thymidine analogues [17]. The purine analogue, [^18^F]FHBG, considered as the gold standard in clinical studies for HSV1-tk reporter gene imaging with PET [18,19] exhibits high abdominal activity due to hepatobiliary elimination [20,21]. When compared with pyrimidines, [^18^F]FHBG shows less sensitivity towards the native HSV1-TK. 

We have previously reported on the synthesis, radiosynthesis and the *in vivo* evaluation of pyrimidine nucleoside analogues in which acyclic 6-(1,3-dihydroxyisobutyl) and 6-(1,3-dihydroxyisobutenyl) side chains have been attached at the C-6 position rather than at the *N*-1 position [22,23,24]. More recently, we have also published results on *N*-Me-[^18^F]FHBT, a *N*-methylated thymine derivative bearing a 6-(1,3-dihydroxyisobutyl) side chain. Compared to [^18^F]FHBG, *N*-Me-[^18^F]FHBT showed a higher background radioactivity in most tissues [25]. Taking into account the aforementioned potential of pyrimidine based nucleosides for the development of novel fraudulent substrates of HSV-1 TK with improved pharmacodynamic and pharmacokinetic profile, we prepared a series of novel C-5 and *N*-1-substituted pyrimidine derivatives [26]. Among these series, a pyrimidine acyclonucleoside bearing a penciclovir (PCV)-like side chain, which was shown to be a substrate of HSV-1 TK, was selected as a lead compound for development as a PET imaging agent for measuring HSV-1 TK expression.

Here we report on the synthesis of unlabeled HHB-5-FEP and the radiosynthesis of its fluorine-18 labeled counterpart, HHB-5-[^18^F]FEP (Figure 1). We further present results of the *in vitro* cellular uptake and the *in vivo* evaluation of HHB-5-[^18^F]FEP. A direct comparison of the small animal PET imaging and biodistribution for HHB-5-[^18^F]FEP and [^18^F]FHBG, as the most commonly used HSV1-TK imaging agent, is also presented. 

## 2. Results and Discussion

### 2.1. Synthesis of the Reference HHB-5-FEP *(**7**)* and Precursor ***8***

**Scheme 1:**
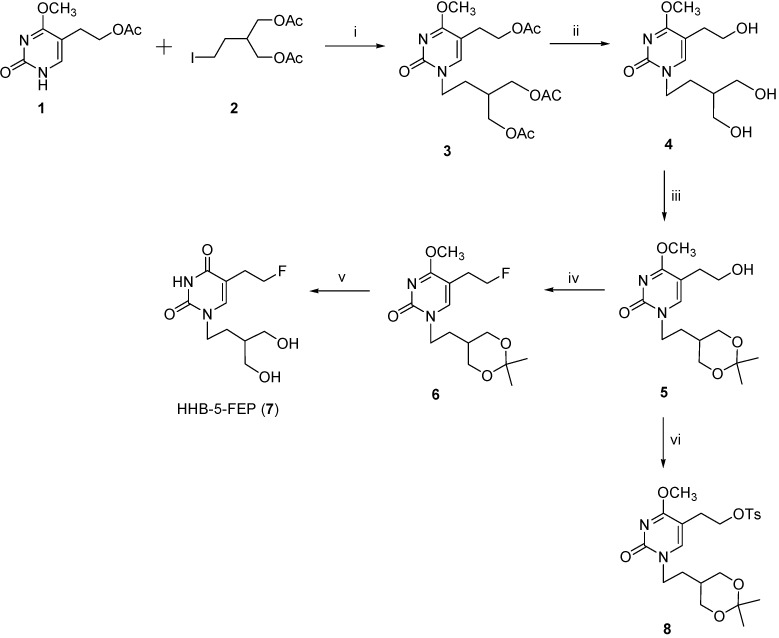
Synthesis of reference compound HHB-5-FEP (**7**) and precursor **8**.

The target compound, *N-*1-[4-hydroxy-3-(hydroxymethyl)butyl]-5-(2-fluoroethyl)pyrimidin-2,4-dione (HHB-5-FEP, **7**) was prepared as outlined in Scheme 1. 

*Reagents and conditions*: (**i**) K_2_CO_3_, DMF, 20 min; (**ii**) 0.1 M NaOMe/MeOH in MeOH, 1 h; (**iii**) p-TsOH × H_2_O, 2,2-dimethoxypropane, DMF, 2 h; (**iv**) DAST, CH_2_Cl_2_, −40 °C, 3 h; (**v**) Method A. NaI, TMSCl, MeCN, reflux, 10 min; Method B. HCl, reflux, 10 min; (**vi**) TsCl, pyridine, 2 h.

The introduction of penciclovir-like chain at *N*-1 position of pyrimidine scaffold was performed by reaction of 5-(2-acetoxyethyl)-4-methoxypyrimidin-2-one (**1**) with 4-acetoxy-(3-acetoxymethyl)butyl iodide (**2**) to give acyclic C-5-substituted pyrimidine derivative **3**. Acetyl groups in both *N*-1 and C-5 side chains were then removed under basic conditions to give the triol **4** [27]. Protection of the 1,3-diols in penciclovir-like side chain was carried out using *p*-toluenesulfonic acid monohydrate (*p*-TsOH×H_2_O) and 2,2-dimethoxypropane to afford acetonide **5** in 59% chemical yield. Transformation of compound **5** to the fluorinated derivative **6** was achieved in a one-step reaction in 34% yield using diethylaminosulfur trifluoride (DAST) as fluorinating reagent. Initial attempts to prepare compound **7** from **6** using NaI, TMSCl in MeCN (Method A) afforded **7** in a somewhat lower yield (24%). Besides the formation of several by-products, purification of **7** also proved tedious. Method B which involves the use of concentrated acid provided target compound **7** in an optimal 29% yield. 

In order to synthesize tosylate precursor **8** for the radiosynthesis of HHB-5-[^18^F]FEP, 5-(2-hydroxyethyl)pyrimidine derivative **5** was treated with *p*-toluenesulfonyl chloride in pyridine to give compound **8** in 68% yield (Scheme 1). The identities of all the synthesized compounds were confirmed by MS and NMR-spectroscopy.

### 2.2. Radiosynthesis of HHB-5-[^18^F]FEP ([^18^F]***7***)

**Scheme 2:**
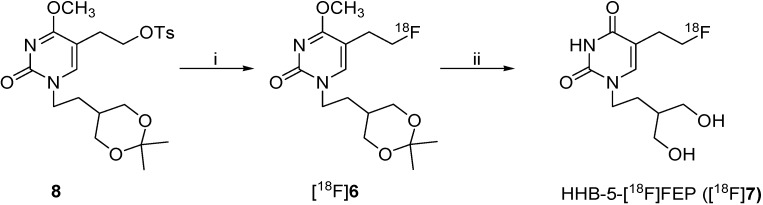
Radiosynthesis of HHB-5-[^18^F]FEP

The radiosynthesis of [^18^F]**7** was carried out using a two-step procedure and consisted of [^18^F]fluorination and cleavage of the protecting groups (Scheme 2). Nucleophilic substitution of the tosyl group by [^18^F]fluoride was performed at 100 °C using MeCN as solvent. A maximal incorporation yield of 60% was accomplished for [^18^F]**6** after a reaction time of 8 min. Cleavage of the protecting groups under acidic conditions at 100 °C afforded HHB-5-[^18^F]FEP in quantitative radiochemical yield.

*Reagents and conditions*: (**i**) [^18^F]KF-K_2.2.2_, MeCN, 100 °C, 8 min; (**ii**) 37% HCl, 100 °C, 10 min.

HHB-5-[^18^F]FEP was purified by a semipreparative radio-HPLC within 25 min (*t*_R_ = 21.08 min) and formulated for *in vitro* and *in vivo* studies. The radiochemical yield was 45% ± 4, (n = 4) decay corrected. The total amount of radioactivity at the end of the synthesis (EOS) was up to 7.36 GBq in a radiochemical purity of > 99%. The specific activity ranged between 50 and 135 GBq/µmol after a total synthesis time of approx. 90 min. The chemical identity of HHB-5-[^18^F]FEP was confirmed by coinjection with the non-radiolabeled reference compound HHB-5-FEP (**7**). 

### 2.3. Cell Uptake Studies

**Figure 2:**
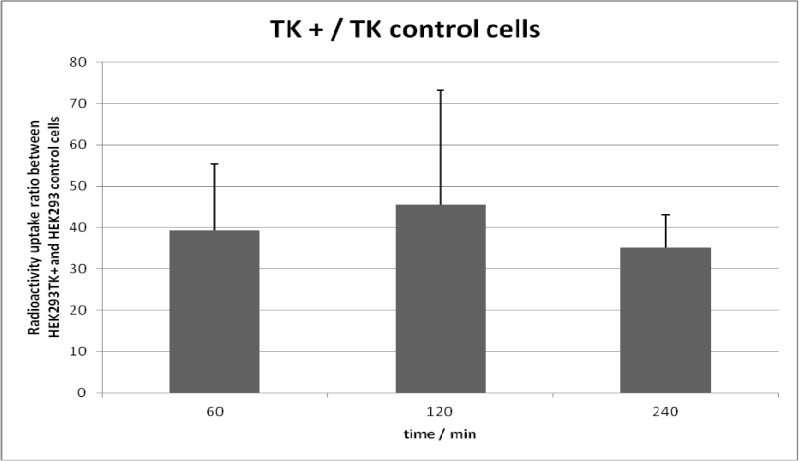
Uptake ratios of HHB-5-[^18^F]FEP in HEK293TK+ and wild type cells.

The *in vitro* uptake of HHB-5-[^18^F]FEP was performed on HEK293TK+ cells and compared to wild type HEK293 cells. The uptake of HHB-5-[^18^F]FEP was at all time points (60, 120, 240 min) higher in HEK293TK+ cells than in control cells. For the investigated time points, the ratio of radioactivity uptake in HEK293TK+ and HEK293 cells was 35–41-fold higher than in wild type cells (Figure 2). This favorable *in vitro* properties encouraged the further *in vivo* testing of HHB-5-[^18^F]FEP. 

### 2.4. Small Animal PET Imaging with HHB-5-[^18^F]FEP and [^18^F]FHBG

**Figure 3:**
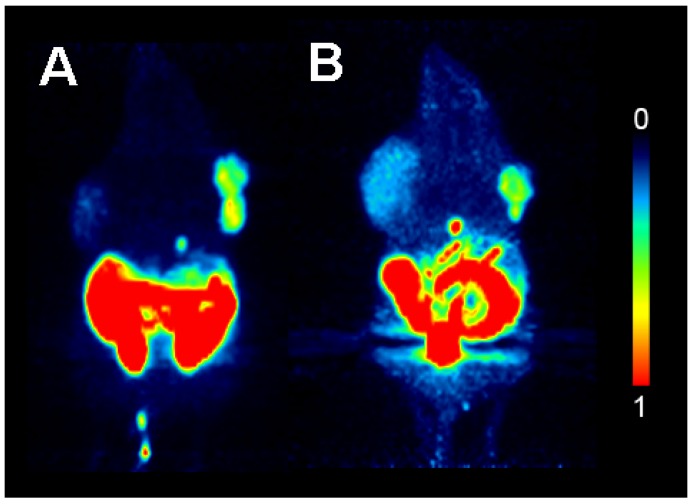
Maximum intensity projections of whole body PET scans of 30 min duration (60–90 min p.i.) performed with HEK293 control tumor bearing mice (left shoulder) and HEK293TK+ (right shoulder) xenografts. (**A**) Mouse (24.3 g) injected with [^18^F]FHBG (17.98 MBq); (**B**) Mouse (28.4 g) injected with HHB-5-[^18^F]FEP (8.47 MBq).

Static whole body (two beds, 60–90 min) PET images of xenograft-bearing mice after i.v. injection of [^18^F]FHBG and HHB-5-[^18^F]FEP are shown in Figure 3. 

### 2.5. Biodistribution

Similar to the *in vitro* cell uptake studies, a higher uptake of HHB-5-[^18^F]FEP in TK-positive xenograft was observed compared to HEK293 control xenograft. The SUV_PET_ for the TK-positive xenograft was 0.32. Uptake in the control xenograft was slightly higher (SUV_PET_ = 0.17) compared to background activity (SUV_PET_ = 0.08). Compared to [^18^F]FHBG, HHB-5-[^18^F]FEP revealed a higher background activity but a lower abdominal radioactivity. [^18^F]FHBG showed similar imaging characteristics as previously shown [28]. The SUV_PET_ for HHB-5-[^18^F]FEP was higher than the SUV_PET_ for [^18^F]FHBG, however, due to the higher background activity of HHB-5-[^18^F]FEP, a lower TK+/control ratio was obtained (Table 1). 

**Table 1:** Biodistribution (SUV_biodis_) and PET data (SUV_PET_) of [^18^F]FHBG and HHB-5-[^18^F]FEP in xenograft-bearing mice at 95 min after radiotracer injection.

Tissue	[^18^F]FHBG			HHB-5-[^18^F]FEP		
	SUV_biodis_	SUV_PET_	Ratio SUV_biodis_	SUV_biodis_	SUV_PET_	Ratio SUV_biodis_
Xenograft TK+	0.24 ± 0.07	0.22 ± 0.02	TK+/contro l5.7 ± 1.9	0.37 ± 0.15	0.32 ± 0.06	TK+/control 3.4 ± 1.1
Xenograft control	0.04 ± 0.00	0.09 ± 0.004		0.11 ± 0.01	0.17 ± 0.02	
Blood	0.03 ± 0.02			0.07 ± 0.04		
Spleen	0.08 ± 0.08			0.05 ± 0.00		
Liver	0.16 ± 0.19			0.06 ± 0.00		
Kidney	0.12 ± 0.06			0.25 ± 0.09		
Lung	0.03 ± 0.01			0.05 ± 0.00		
Bone	0.04 ± 0.03			0.05 ± 0.01		
Heart	0.04 ± 0.05			0.04 ± 0.01		
Brain	0.002 ± 0.00			0.01 ± 0.00		
Stomach w. cont.	0.02 ± 0.01			0.05 ± 0.01		
Intestine w. cont.	1.78 ± 0.04			0.78 ± 0.05		
Pancreas	0.03 ± 0.01			0.06 ± 0.02		
Muscle	0.02 ± 0.00	0.04 ± 0.01		0.08 ± 0.02	0.08 ± 0.002	
Thyroid	0.02 ± 0.01			0.12 ± 0.11		
Gallbladder	0.01 to 3.6			1.5 to 3.5		
Urine	15 to 29			13 to 182		

The region with the highest uptake radioactivity was the abdomen followed by the gallbladder and the TK-positive xenograft. Table 1 summarizes the biodistribution data of [^18^F]FHBG and HHB-5-[^18^F]FEP in xenograft-bearing mice at 95 min after radiotracer injection. SUV_biodis_ data are the mean ± standard deviation of three animals for [^18^F]FHBG and HHB-5-[^18^F]FEP. Radioactivity accumulation for [^18^F]FHBG and HHB-5-[^18^F]FEP in TK-positive xenografts was significantly higher than in the control xenografts (*p* = 0.004 and 0.033, respectively, Student’s t-Test). The uptake ratio of TK+ to control xenograft was 5.7 ± 1.9 for [^18^F]FHBG (n = 3) and 3.4 ± 1.1 for HHB-5-[^18^F]FEP (n = 3). The distribution patterns of the two tracers were similar, however, HHB-5-[^18^F]FEP exhibited lower radioactivity levels in the intestine, liver and spleen and higher activity in the muscles, blood, kidneys, thyroid gland and brain area. High accumulation of radioactivity in kidneys, gallbladder and urine suggested renal excretion pathway and resulted in lower intestine activity when compared to [^18^F]FHBG. Both radiotracers showed very low bone uptake indicating negligible *in vivo* defluorination. 

## 3. Experimental

### 3.1. General

Melting points (uncorrected) were determined with Kofler micro hot-stage (Reichert, Wien, Austria). Precoated Merck (Darmstadt, Germany) silica gel 60F-254 plates were used for thin layer chromatography and the spots were detected under UV light (254 nm). Column chromatography was performed using silica gel (0.063–0.2 mm) Fluka (Buchs, Switzerland); glass column was slurry-packed under gravity. ^1^H- and ^13^C-NMR spectra were acquired on a Bruker 300 MHz NMR spectrometer (Bruker Biospin, Rheinstetten, Germany). All data were recorded in DMSO-d_6_ at 298 K. Chemical shifts were referenced to the residual solvent signal of DMSO at 2.50 ppm for ^1^H and 39.50 ppm for ^13^C. Individual resonances were assigned on the basis of their chemical shifts, signal intensities, multiplicity of resonances and HH coupling constants. Mass spectra were recorded on an Agilent 6410 instrument (Agilent Technologies, Wilmington, DE, USA) equipped with electrospray interface and triple quadrupole analyzer (LC/MS/MS). Reagents and solvents were purchased from Sigma-Aldrich Chemie GmbH (Buchs, Switzerland), Acros Organics (Geel, Belgium) and VWR International AG (Dietikon, Switzerland) and used without further purification.

Purification of the radiolabeled products was performed using a semi-preparative HPLC system, equipped with a Smartline Pump 1000, Smartline Manager 5000, Smartline UV detector 2500 (Knauer, city, state abbrev if US, country), 3 mL-loop and a GabiStar radiodetector (Raytest, Straubenhardt, Germany). A semi-preparative HPLC column with precolumn (Phenomenex, Gemini, C18, 110 Å, 5 µm, 250 × 10 mm) was used and UV absorption was monitored at 254 nm wavelength. For the purification of HHB-5-[^18^F]FEP, a 20 mM sodium phosphate buffer at pH 7.4, containing 5% EtOH was prepared (11.29 mmol/L NaH_2_PO_4_·H_2_O and 38.71 mmol/L Na_2_HPO_4_ × 2H_2_O). For the purification of [^18^F]FHBG, a mobile phase consisting of 5% EtOH in H_2_O was used. 

For quality control, an aliquot of the formulated solution was injected into an analytical Agilent 1100 series HPLC system, equipped with a 100 µL-loop and a GabiStar radiodetector (Raytest). An analytical HPLC column (Phenomenex, Gemini, C18, 110 Å, 5 µm, 250 × 4.6 mm) was used at a flow rate of 1 mL/min. For the analysis of HHB-5-[^18^F]FEP, a gradient method starting from 100% A to 50% B within 30 min was used (A = H_2_O, B = MeCN) and for [^18^F]FHBG, a gradient method starting from 100% A to 15% B within 15 min was used (A = H_2_O, B = MeCN). UV absorption was recorded at 254 nm wavelength. The radiosynthesis of ^18^F]FHBG was performed as previously reported. For the analysis of [^18^F]FHBG, an isocratic method of 97% A and 3% B was used (A = 0.1% TFA in H_2_O, B = MeCN). 

Specific activity was calculated based on the integrated UV peak by using a calibration curve, derived from different concentrations of the reference compound HHB-5-FEP and FHBG, respectively.

### 3.2. Cell Lines

#### 3.2.1. Cell Culture

Cell uptake and internalization experiments were performed as previously described [25]. In brief, HEK293 human embryonic kidney cells and HEK293 stable transfected with nonmutant HSV1-tk (HEK293TK+ cells) were cultured in high glucose DMEM media supplemented with 10% FBS and 1% Penicillin/Streptomycin. Cells were grown in humidified atmosphere with 5% CO_2_ at 37 °C. Thymidine kinase expression in the HEK293TK+ cells was maintained with 0.3 mg/mL G418 in the culture medium and was routinely verified by visualization of the cotransfected RFP with a fluorescence microscope.

#### 3.2.2. Cell Uptake

HEK293TK+ and HEK293 control cells were seeded in 12-well culture plates (8 × 10^5^ cells per well) in DMEM supplemented with 10% FBS. After 24 h, when cultures reached 80% confluence, cells were incubated for 30, 60, and 240 min with medium (1 mL) containing 130 kBq HHB-5-[^18^F]FEP per well. At the end of the incubation, cells were washed twice with PBS and detached with 0.25% trypsin (0.3 mL). The cells were resuspended in culture medium (0.7 mL), sedimented and lysed in lysis buffer (0.5 mL, 0.0625 M Tris, 2% sodium dodecyl sulfate, 7% glycerol, pH 6.8). The radioactivity of the cell lysate and the combined incubation medium were measured in a gamma counter (Wizard; PerkinElmer). Radioactivity of the cell lysates was normalized to total protein determined in cell lysate (50 μL) with the *DC*^™^ Protein Assay Kit I (BioRad, Hercules, CA). Data were expressed as percent accumulated activity/μg protein ((dpm cells × 100%/(dpm cells + dpm medium))/μg protein) and uptake ratios (dpm HEK293TK+/dpm HEK293 control), respectively [29]. 

### 3.3. Animals

All animal experiments were approved by the local veterinarian department and complied with Swiss and local laws on animal protection. Six week-old female NMRI nude mice were purchased from Charles River Laboratories (Sulzfeld, Germany). Under 2-3% isoflurane anesthesia, animals were injected subcutaneously with 5 × 10^6^ cells in Matrigel (100 µL). Transfected HEK293TK+ cells were injected on the right side of the shoulder region, control HEK293 cells on the left side. Xenograft growth and body weight were monitored regularly. Experiments were conducted when the xenografts reached a volume of 1–2 cm^3^, which was approx. four weeks after inoculation.

#### 3.3.1. *In Vivo* PET Scan

For PET imaging, eXplore VISTA PET/CT tomograph (Sedecal/GE Healthcare, Madrid, Spain) was used. Nude mice, bearing HEK293TK+ xenografts on the right shoulder and HEK293 xenografts (control) on the left shoulder were injected with HHB-5-[^18^F]FEP or [^18^F]FHBG formulations (100 μL per injection) via lateral tail vein injection (*t* = 0). After injection of the radiotracer, mice were anesthetized by inhalation of isoflurane in an air/oxygen mixture approximately 5 min prior to PET data acquisition and scanned as described previously [30]. In dynamic PET mode, one animal was scanned from 0–90 min. For the second animal, data were acquired from 60–150 min p.i. For static PET scans, mice were scanned from 60–90 min. as it revealed to be the optimal time frame. After acquisition, PET data were reconstructed and fused datasets of PET and CT were analyzed with PMOD software (version 3.4).

#### 3.3.2. *Ex Vivo* Biodistribution Studies of [^18^F]FHBG and of HHB-5-[^18^F]FEP

To perform *ex vivo* biodistribution studies of HHB-5-[^18^F]FEP and [^18^F]FHBG, HEK293 control and HEK293TK+ xenograft bearing nude mice were euthanized immediately after the static PET experiments, which was 95 min post tracer application (n = 3). Organs and tissue samples were weighed and the radioactivity determined in a gamma counter. Decay corrected radioactivity was expressed in analogy to the SUV (standardized uptake values) as the ratio of the detected activity per gram tissue and the injected dose per gram body weight (SUV_biodis_).

### 3.4. Procedures for the Preparation of Compounds

*N*-1-[4-Acetoxy-3-(acetoxymethyl)butyl]-5-(2-acetoxyethyl)-4-methoxypyrimidin-2-one (**3**) and *N*-1-[4-hydroxy-3-(hydroxymethyl)butyl]-5-(2-hydroxyethyl)-4-methoxypyrimidin-2-one (**4**) were prepared according to a previously published procedure [27]. 

*N-1-[2-(2,2-Dimethyl-1,3-dioxane-5-yl)ethyl]-5-(2-hydroxyethyl)-4-methoxypyrimidin-2-one* (**5**). To a stirred solution of compound **4** (726 mg, 2.67 mmol) in dry DMF (9 mL), 2,2,-dimethoxypropane (0.61 mL, 3.89 mmol) and *p*-toluenesulfonic acid monohydrate (15.17 mg, 0.08 mmol) were added. The stirring was continued for 2 h at room temperature. The reaction mixture was then neutralized by triethylamine. The solvent was removed under reduced pressure and the residue was purified by column chromatography on silica gel (CH_2_Cl_2_-CH_3_OH = 10:1) to give compound **5** as white crystals (494.7 mg, 59%, mp: 120–121 °C). ^1^H-NMR: (δ) 7.78 (1H, s, H-6), 4.60 (1H, t, OH, *J* = 4.5 Hz), 3.83 (3H, s, OCH_3_), 3.72–3.82 (4H, m, H-4'', H-2'), 3.44–3.57 (4H, m, H-4'', H-1''), 2.41 (2H, t, H-1', *J* = 6.7 Hz), 1.58–1.70 (1H, m, H-3''), 1.53 (2H, q, H-2'', *J* = 7.1 Hz), 1.33 (3H, s, CH_3_), 1.27 (3H, s, CH_3_) ppm. ^13^C-NMR: (δ) 170.0 (C-4), 155.7 (C-2), 147.2 (C-6), 104.6 (C-5), 97.5 (C-5''), 63.9 (C-4''), 61.9 (C-2'), 60.0 (C-4''), 54.3 (OCH_3_), 47.8 (C-1''), 41.3 (C-3''), 30.0 (C-1'), 28.3 (C-2''), 27.1 (CH_3_), 21.6 (CH_3_) ppm. MS (ESI): *m/z* = 313.2 ([*M*+H]^+^).

*N-1-[2-(2,2-Dimethyl-1,3-dioxane-5-yl)ethyl]-5-(2-fluoroethyl)-4-methoxypyrimidin-2-one* (**6**)*.* A solution of compound **5** (296.7 mg, 0.95 mmol) in anhydrous CH_2_Cl_2_ (7 mL) was cooled to −68 °C and diethylaminosulfur trifluoride (DAST) (0.27 mL, 2.04 mmol) was added under argon atmosphere. The reaction mixture was stirred at −40 °C for 3 h and then allowed to warm up to room temperature. The solvent was evaporated and the residue chromatographed (CH_2_Cl_2_-CH_3_OH = 15:1). Compound **6** was isolated as a colourless oil (100.2 mg, 34%). ^1^H-NMR: (δ) 7.89 (1H, s, H-6), 4.52 (2H, dt, H-2', *J_H-F_* = 47.1 Hz, *J_H-H_* = 6.3 Hz), 3.84 (3H, s, OCH_3_), 3.74–3.80 (2H, m, H-4''), 3.50–3.55 (4H, m, H-4'', H-1''), 2.64–2.72 (2H, dt, H-1', *J_H-F_* = 23.4 Hz, *J_H-H_* = 6.3 Hz), 1.61–1.67 (1H, m, H-3''), 1.51–1.55 (2H, m, H-2''), 1.32 (3H, s, CH_3_), 1.27 (3H, s, CH_3_) ppm. MS (ESI): *m/z* 315.1 ([*M*+H]^+^).

*N-1-[4-Hydroxy-3-(hydroxymethyl)butyl]-5-(2-fluoroethyl)pyrimidin-2,4-dione* (HHB-5-FEP, **7**)*.*

*Method A*. To a solution of compound **6** (40 mg, 0.13 mmol) and MeCN (2 mL), trimethylsilyl chloride (TMSCl) (0.05 mL, 0.39 mmol) and NaI (39.5 mg, 0.26 mmol) were added under argon atmosphere. The mixture was stirred at reflux for 10 min, the solvent was evaporated under reduced pressure and the residue chromatographed (EtOAc-CH_3_OH = 10:1). Compound **7** was obtained as yellow oil (8.0 mg, 24%).

*Method B*. Compound **6** (37.3 mg, 0.12 mmol) was dissolved in concentrated 37% HCl (1.6 mL) and the mixture was stirred at reflux for 10 min. Then, it was neutralized by the addition of 6 M NaOH. The salt was filtered of, the solvent was evaporated under reduced pressure and the residue chromatographed (CH_2_Cl_2_-CH_3_OH = 10:1). Compound **7** was obtained as colourless oil (9.0 mg, 29%). ^1^H-NMR: (δ) 11.24 (1H, s, NH), 7.58 (1H, s, H-6), 4.50 (2H, dt, H-2', *J_H-F_* = 47.3 Hz, *J_H-H_* = 6.3 Hz), 4.37 (2H, bs, OH), 3.70 (2H, t, H-1'', *J* = 7.6 Hz), 3.39–3.42 (2H, m, H-4''), 3.33–3.35 (2H, m, H-4''), 2.58 (2H, t, H-1', *J_H-F_* = 23.1 Hz, *J_H-H_* = 6.3 Hz), 1.55 (2H, q, H-2'', *J* = 7.2 Hz), 1.45–1.49 (1H, m, H-3'') ppm. ^13^C-NMR: (δ)163.8 (C-4), 150.6 (C-2), 143.1 (C-6), 115.3 (C-5), 82.2, 81.1 (C-2’, *J* = 164.7 Hz), 61.4 (C-4''), 45.9 (C-1''), 40.7 (C-3''), 27.8 (C-2''), 27.5, 27.3 (C-1', *J* = 21.1 Hz) ppm. MS (ESI): *m/z* = 261.2 ([*M*+H]^+^).

*N-1-[2-(2,2-Dimethyl-1,3-dioxane-5-yl)ethyl]-5-[2-((p-toluenesulfonyl)oxy)ethyl]-4-methoxy-pyrimidin-2-one* (**8**). *p*-Toluenesulfonyl chloride (TsCl, 357 mg, 1.87 mmol) dissolved in pyridine (3 mL) was added to a cooled solution (0 °C) of compound **5** (100 mg, 0.32 mmol) in anhydrous pyridine (5 mL). The reaction mixture was stirred at 0 °C for 1 h then allowed to warm to room temperature and additionally stirred for 1 h. Ethyl acetate (50 mL) was added to the reaction mixture and the organic layer was extracted three times with water (2 × 30 mL). The aqueous washings were extracted again with ethyl acetate (2 × 30 mL) and the combined organic layer was dried over anhydrous MgSO_4_. The drying agent was filtered off, the solvent was removed by rotary evaporation and the residue chromatographed on silica gel (CH_2_Cl_2_-CH_3_OH = 15:1). Compound **8** was isolated as a colourless oil (102.2 mg, 68%). ^1^H-NMR: (δ) 7.77 (1H, s, H-6), 7.65–7.69 (m, 2H, Ph), 7.39–7.43 (2H, m, Ph), 4.14 (2H, t, H-2', *J* = 6.3 Hz), 3.76–3.82 (2H, m, H-4''), 3.69–3.73 (within OCH_3_ signal, H-1''), 3.71 (3H, s, OCH_3_), 3.51–3.56 (2H, m, H-4''), 2.57 (2H, t, H-1', *J* = 6.3 Hz), 2.41 (3H, s, CH_3_), 1.60–1.67 (1H, m, H-3''), 1.52 (2H, q, H-2'' *J* = 7.3 Hz), 1.34 (3H, s, CH_3_), 1.28 (3H, s, CH_3_) ppm. ^13^C-NMR: (δ )168.9 (C-4), 155.0 (C-2), 147.4 (C-6), 145.0 (Ph-4), 132.1 (Ph-1), 130.0 (Ph-3), 127.3 (Ph-2), 101.4 (C-5), 97.2 (C-5''), 68.6 (C-4''), 63.3 (C-2'), 61.5 (C-4''), 53.7 (OCH_3_), 46.3 (C-1''), 40.1 (C-3''), 31.2 (CH_3_), 27.7 (C-1'), 26.4 (CH_3_), 25.9 (C-2''), 21.4 (CH_3_) ppm. MS (ESI): *m/z* = 467.3 ([*M*+H]^+^).

Production of Dried [^18^F]fluoride

No-carrier-added [^18^F]fluoride was obtained by irradiation of a liquid target filled with isotopically enriched [^18^O]H_2_O (1900 µL, 97%, Cambridge Isotope Laboratories, Burgdorf, Switzerland), by an 18 MeV proton beam on a 18/9 cyclotron (IBA, Ottignies-Louvain-la-Neuve, Belgium). After the end of bombardment radioactivity (31–54 GBq) was transferred to a synthesis hot cell by a continuous stream of helium. The aqueous [^18^F]fluoride solution was trapped on a Sep-Pak Light Accell Plus carb QMA cartridge (Waters, Baden, Switzerland) without preconditioning. Elution was performed by using a solution of K_2_CO_3_ (1.8 mg, 13 μmol) and Kryptofix K_2.2.2_ (10 mg, 26.6 μmol) in a mixture of H_2_O (0.6 mL) and MeCN (1.4 mL). The eluate was collected in a sealed Wheaton reactor (5 mL) and the solvent was evaporated at 90 °C under reduced pressure and a gentle stream of nitrogen for 10 min. Subsequently, MeCN (1 mL) was added three times and evaporated to dryness within 3 min at 90 °C. Finally, full vacuum without nitrogen stream was applied for 5 min at 90 °C. 

*Radiosynthesis of N-1-[4-hydroxy-3-(hydroxymethyl)butyl]-5-(2[^18^F]fluoroethyl)pyrimidine-2,4-dione (HHB-5-[^18^F]FEP, [^18^]**7**).*

For the preparation of [^18^F]**7**, the precursor (**8**) (4 mg, 8.6 µmol) was dissolved in anhydrous MeCN (300 µL) and added to the azeotropically dried [^18^F]fluoride-cryptate complex (25–42 GBq) (Scheme 2). The solution was heated at 100 °C for 8 min and was allowed to cool down for 5 min. MeCN (1 mL) was added and the solution was passed through a Sep-Pak light silica cartridge (Waters, preconditioned with 5 mL Et_2_O) to remove unreacted [^18^F]fluoride and kryptofix/carbonate salts from [^18^F]**6**. The reactor was additionally rinsed with MeCN (1 mL) and the organic phase was also passed through the Sep-Pak light silica cartridge. The combined MeCN phase was evaporated to dryness under reduced pressure and a gentle stream of nitrogen at 90 °C. For deprotection, concentrated hydrochloric acid (0.5 mL) was added to the reaction vessel and the solution was heated for 10 min at 100 °C to give [^18^F]**7**. After cooling for 5 min, 5 M NaOH (1 mL) was added to neutralize the acidic solution and 0.6 M PBS (1.0 mL) and water (0.5 mL) were added for dilution to a total volume of 3 mL. The final radiolabeled product was purified by using a semi-preparative radio-HPLC. The product fraction was collected and passed through a sterile filter into a sterile pyrogen-free vial ready to use for further experiments. The specific activity ranged between 50 and 135 GB q/µmol. A chromatogram of the HPLC analysis of the formulated radiotracer is available in the Supplementary Materials.

Radiosynthesis of [^18^F]FHBG ([^18^F]**11**).

For preparation of [^18^F]FHBG, the tosyl precursor (3 mg, 3.15 µmol) was dissolved in anhydrous MeCN (400 µL) and added to the azeotropically dried [^18^F]fluoride-cryptate complex (30–38 GBq). The solution was heated at 115 °C for 20 min and was allowed to cool down for 10 min. A mixture of 15% MeOH in CH_2_Cl_2_ (1 mL) was added and the solution was passed through a Sep-Pak light silica (preconditioned with 5 mL Et_2_O). The reactor was additionally rinsed with 15% MeOH in CH_2_Cl_2_ (3 mL) and the solution was also passed through the Sep-Pak light silica cartridge. The organic phase was evaporated to dryness under reduced pressure and a gentle stream of nitrogen at 90 °C. For deprotection, 1 M hydrochloric acid (0.6 mL) was added to the reaction vessel and the solution was heated for 10 min at 115 °C to give [^18^F]FHBG. After cooling for 5 min, 1 M NaOH (0.6 mL) was added to neutralize the acidic solution. Then, 0.6 M PBS (1 mL) and water (0.8 mL) were added for dilution to a total volume of 3 mL. The radioproduct was purified by using a semi-preparative radio-HPLC. The product fraction was collected and passed through a sterile filter into a sterile pyrogen-free vial.

## 4. Conclusions

The novel *N*-acyclic 5-(2-fluoroethyl)pyrimidine nucleoside analogue, HHB-5-FEP, was synthesized in a five-step reaction sequence starting from 5-(2-acetoxyethyl)-4-methoxypyrimidin-2-one. Synthesis of its ^18^F labeled structural analogue, HHB-5-[^18^F]FEP ([^18^F]**7**), was accomplished in two steps by nucleophilic substitution on a tosyl leaving using [^18^F]fluoride-cryptate complex and subsequent removal of the 4-methoxy and isopropylidene protecting groups under acidic conditions. The overall maximal radiochemical yield for HHB-5-[^18^F]FEP was significantly higher (45%) than that for the routinely used [^18^F]FHBG (16%). 

Cell uptake studies of HHB-5-[^18^F]FEP showed 35–41-fold higher accumulation of radioactivity in TK+ cells than in control cells. HHB-5-[^18^F]FEP in tumor bearing mice clearly visualized HSV1-tk expressing tumors but the contrast between transduced and non-transduced xenografts was higher for [^18^F]FHBG due to the low background radioactivity. A clear advantage of HHB-5-[^18^F]FEP is the lower abdominal radioactivity when compared to [^18^F]FHBG. HHB-5-[^18^F]FEP may thus allow the monitoring of HSV1-tk expression in areas close to the abdominal region which otherwise would not be possible with [^18^F]FHBG. Although pyrimidine acyclonucleoside HHB-5-[^18^F]FEP was not superior to [^18^F]FHBG, we have successfully demonstrated its potential for the *in situ* monitoring of HSV1-tk expression. 

## Supplementary Materials

Supplementary Materials containing ^1^H- and ^13^C-NMR spectra of compounds **4**, **5**, **7** and **8** and HPLC chromatogram of [^18^F]**7** coinjected with the nonradioactive reference compound **7** can be accessed at: http://www.mdpi.com/1420-3049/18/7/8535/s1.

## References

[1] Rosé C., Dose J., Avril N. (2002). Positron emission tomography for the diagnosis of breast cancer. Nucl. Med. Commun..

[2] Vasselle H., Grierson J., Muzi M., Pugsley J.M., Schmidt R.A., Rabinowitz P., Peterson L.M., Vallie`res E., Wood D.E. (2002). *In vivo* validation of 3'-deoxy-3'-[^18^F]fluorothymidine ([^18^F]FLT) as a proliferation imaging tracer in humans: co-relation of ^18^F-FLT uptake by positron emission tomography with Ki-67 immunohistochemistry and flow cytometry in human lung tumors. Clin. Cancer Res..

[3] Soghomonyan S., Hajitou A., Rangel R., Trepel M., Pasqualini R., Arap W., Gelovani J.G., Alauddin M.M. (2007). Molecular PET imaging of HSV1-tk reporter gene expression using [^18^F]FEAU. Nat. Protoc..

[4] Yeh H.H., Ogawa K., Balatoni J., Mukhapadhyay U., Pal A., Gonzales-Lepera C., Shavrina A., Soghomonyana S., Flores II L., Younga D. (2002). Molecular imaging of active mutant L858R EGFR kinase expressing non small cell lung carcinomas using PET/CT with [^18^F]F-PEG6-IPQA. PNAS.

[5] Kostakoglu L., Goldsmith S.J. (2003). [^18^F]-FDG PET evaluation of the response to therapy for lymphoma and for breast, lung, and colorectal carcinoma. J. Nucl. Med..

[6] Paolillo V., Yeh H.H., Mukhopadhyay U., Gelovani J.G., Alauddin M.M. (2011). Improved detection and measurement of low levels of [^18^F]fluoride metabolized from [^18^F]-labeled pyrimidine nucleoside analogues in biological samples. Nucl. Med. Biol..

[7] Brader P., Wong R.J., Horowitz G., Gil Z. (2012). Combination of PET imaging with viral vectors for identification of cancer metastases. Adv. Drug Deliv. Rev..

[8] Alauddin M.M., Conti P.S., Fissekis J.D. (2002). Synthesis of [^18^F]-labeled 2'-deoxy-2'-fluoro-5-methyl-1-b-D-arabinofuranosyluracil) [^18^F]-FMAU. J. Labelled Compd. Radiopharm..

[9] Mangner T.J., Klecker R.W., Anderson L., Shields A.F. (2003). Synthesis of 2'-deoxy-2'-[^18^F]fluoro-beta-D-arabinofuranosyl nucleosides, [^18^F]FAU, [^18^F]FMAU, [^18^F]FBAU and[^18^F]FIAU, as potential PET agents for imaging cellular proliferation. Synthesis of [^18^F]labelled FAU, FMAU, FBAU, FIAU. Nucl. Med. Biol..

[10] Buursma A.R., Rutgers V., Hospers G.A., Mulder N.H., Vaalburg W., de Vries E.F.J. (2006). ^18^FFEAU as a radiotracer for herpes simplex virus thymidine kinase gene expression: *in vitro* comparison with other PET tracers. Nucl. Med. Commun..

[11] Yaghoubi S.S., Couto M.A., Chen C.C., Polavaram L., Cui G., Sen L., Gambhir S.S. (2006). Preclinical safety evaluation of ^18^F-FHBG: a PET reporter probe for imaging herpes simplex virus type 1 thymidine kinase (HSV1-tk) or mutant HSV1-sr39tk’s expression. J. Nucl. Med..

[12] Brust P., Haubner R., Friedrich A., Scheunemann M., Anton M., Koufaki O.N., Hauses M., Noll S., Noll B., Haberkorn U. (2001). Comparison of [^18^F]FHPG and [^124/125^I]FIAU for imaging herpes simplex virus type1 thymidine kinase gene expression. Eur. J. Nucl. Med..

[13] Alauddin M.M., Shahinian A., Park R., Tohme M., Fissekis J.D., Conti P.S. (2007). In vivo evaluation of 2'-deoxy-2'-[(^18^)F]fluoro-5-iodo-1-beta-D-arabinofuranosyluracil ([^18^F]FIAU) and 2'-deoxy-2'-[^18^F]fluoro-5-ethyl-1-beta-D-arabinofuranosyluracil ([^18^F]FEAU) as markers for suicide gene expression. Eur. J. Nucl. Med. Mol. Imaging.

[14] Miyagawa T., Gogiberidze G., Serganova I., Cai S., Balatoni J.A., Thaler H.T., Ageyeva L., Pillarsetty N., Finn R.D., Blasberg R.G. (2008). Imaging of HSV-tk Reporter gene expression: comparison between[^18^F]FEAU, [^18^F]FFEAU, and other imaging probes. J. Nucl. Med..

[15] Gambhir S.S., Herschman H.R., Cherry S.R., Barrio J.R., Satyamurthy N., Toyokuni T., Phelps M.E., Larson S.M., Balatoni J., Finn R. (2000). Imaging transgene expression with radionuclide imaging technologies. Neoplasia.

[16] De Clercq E. (2004). Antivirals and antiviral strategies. Nat. Rev. Microbiol..

[17] Huang H.-L, Chiang L.-W., Chen J.-R., Yang W.K., Jeng K.-C., Chen J.-T., Duh T.-S., Lin W.-J., Farn S.-S., Chiang C.-S. (2012). Study of [^18^F]FLT and [^123^I]IaraU for cellular imaging in HSV1 tk-transfected murine fibrosarcoma cells: evaluation of the tracer uptake using 5-fluoro, 5-iodo and 5-iodovinyl arabinosyl uridines as competitive probes. Nucl. Med. Biol..

[18] Yaghoubi S.S., Jensen M.C., Satyamurthy N., Budhiraja S., Paik D., Czernin J., Gambhir S.S. (2009). Noninvasive detection of therapeutic cytolytic T cells with ^18^F-FHBG PET in a patient with glioma. Nat. Clin. Pract. Oncol..

[19] Yaghoubi S.S., Gambhir S.S. (2006). PET imaging of herpes simplex virus type 1 thymidine kinase (HSV1-tk) or mutant HSV1-sr39tk reporter gene expression in mice and humans using [^18^F]FHBG. Nat. Protoc..

[20] Tjuvajev J.G., Doubrovin M., Akhurst T., Cai S., Balatoni J., Alauddin M.M., Finn R., Bornmann W., Thaler H., Conti P.S. (2002). Comparison of radiolabeled nucleoside probes (FIAU, FHBG, and FHPG) for PET imaging of HSV1-tk gene expression. J. Nucl. Med..

[21] Yaghoubi S., Barrio J.R., Dahlbom M., Iyer M., Namavari M., Satyamurthy N., Goldman R., Herschman H.R., Phelps M.E., Gambhir S.S. (2001). Human pharmacokinetic and dosimetry studies of [^18^F]FHBG: a reporter probe for imaging herpes simplex virus type-1 thymidine kinase reporter gene expression. J. Nucl. Med..

[22] Raić-Malić S., Johayem A., Ametamey S.M., Batinac S., De Clercq E., Folkers G., Scapozza L. (2004). Synthesis, 18F-radiolabelling and biological evaluations of C-6 alkylated pyrimidine nucleoside analogues. Nucleosides Nucleotides Nucleic Acids.

[23] Johayem A., Raić-Malić S., Lazzati K., Schubiger P.A., Scapozza L., Ametamey S.M. (2006). Synthesis and characterization of a C(6) nucleoside analogue for the *in vivo* imaging of the gene expression of herpes simplex virus type-1 thymidine kinase (HSV1 TK). Chem. Biodiv..

[24] Krištafor S., Novaković I., Gazivoda Kraljević T., Kraljević Pavelić S., Lučin P., Westermaier Y., Pernot L., Scapozza L., Ametamey S.M., Raić-Malić S. (2011). Synthetic Approach to New *N*-methyl Thymine Derivative Comprising Dihydroxyisobutenyl Unit as Ligand for Thymidine Kinase of Herpes Simplex Virus Type 1 (HSV1-TK). Bioorg. Med. Chem. Lett..

[25] Müller U., Martić M., Gazivoda Kraljević T., Krištafor S., Ross T.L., Ranadheera C., Müller A., Born M., Krämer S.D., Raić-Malić S. (2012). Synthesis and evaluation of a C-6 alkylated pyrimidine derivative for the *in vivo* imaging of HSV1-TK gene expression. Nucl. Med. Biol..

[26] Meščić A., Krištafor S., Novaković I., Osmanović A., Müller U., Završnik D., Ametamey S.M., Scapozza L., Raić-Malić S. (2013). C-5 Hydroxyethyl and Hydroxypropyl Acyclonucleosides as Substrates for Thymidine Kinase of Herpes Simplex Virus Type 1 (HSV-1 TK): Syntheses and Biological Evaluation. Molecules.

[27] Meščić A., Glavač D., Osmanović A., Završnik D., Cetina M., Makuc D., Plavec J., Ametamey S.M., Raić-Malić S. (2013). *N* -alkylated and *O* -alkylated regioisomers of 5-(hydroxyalkyl)pyrimidines: Synthesis and structural study. J. Mol. Struct..

[28] Müller U., Ross T.L, Ranadheera C., Slavik R., Müller A., Born M., Trauffer E., Miličević Sephton S., Scapozza L., Krämer S.D., Ametamey S.M. (2013). Synthesis and preclinical evaluation of a new C-6 alkylated pyrimidine derivative as a PET imaging agent for HSV1-tk gene expression. Am. J. Nucl. Med. Mol. Imaging.

[29] Yaghoubi S.S, Gambhir S.S. (2006). Measuring herpes simplex virus thymidine kinase reporter gene expression *in vitro*. Nat. Protoc..

[30] Honer M., Brühlmeier M., Missimer J., Schubiger A.P., Ametamey S.M. (2004). Dynamic imaging of striatal D2 receptors in mice using quad-HIDAC PET. J. Nucl. Med..

