# Supplementary material for: Synthesis and Biological Evaluation of a New Acyclic Pyrimidine Derivative as a Probe for Imaging Herpes Simplex Virus Type 1 Thymidine Kinase Gene Expression
Source: Molecules. 2013 Jul 19;18(7):8535–49. doi: 10.3390/molecules18078535 (PMC6270388; doi:10.3390/molecules18078535)

Supplementary Materials

Figure S1. ^{1}H-NMR spectrum of compound **4**.

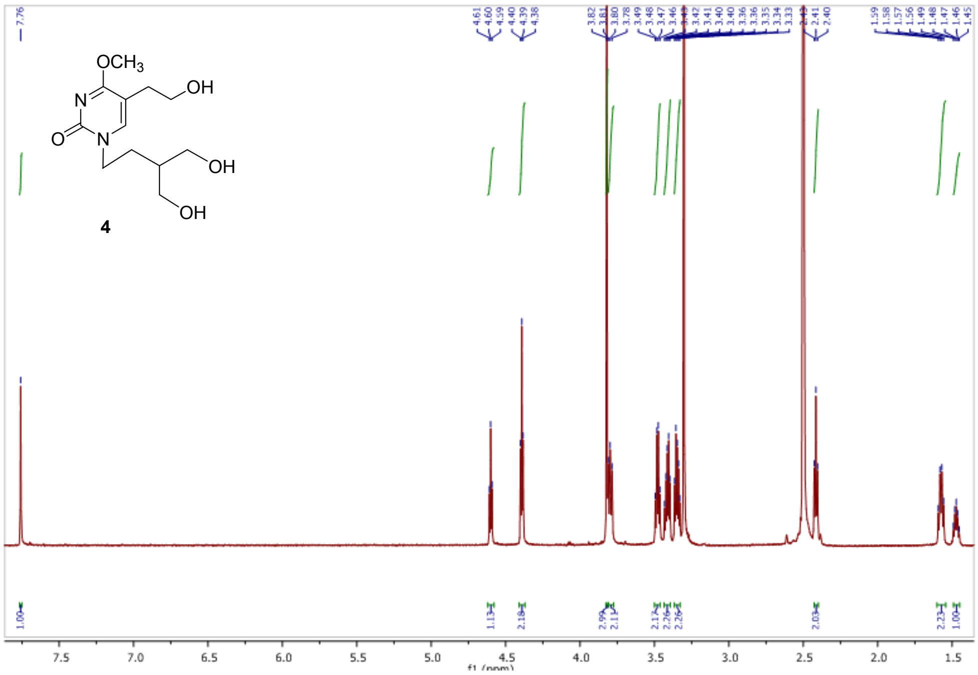

Figure S2. ^{13}C-NMR spectrum of compound **4**.

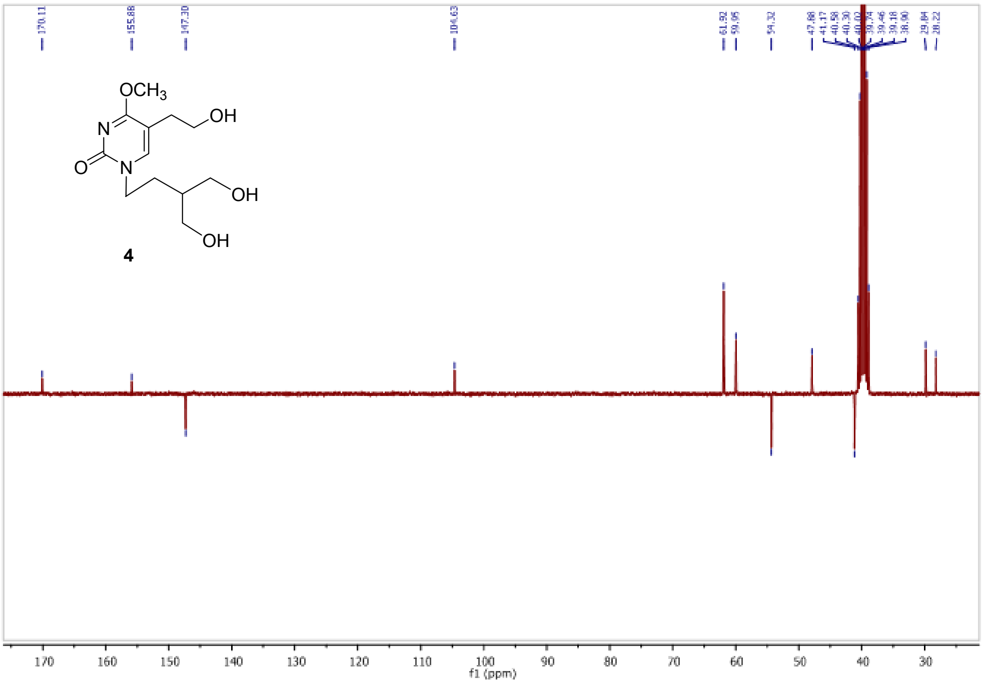

Figure S3. ^{1}H-NMR spectrum of compound **5**.

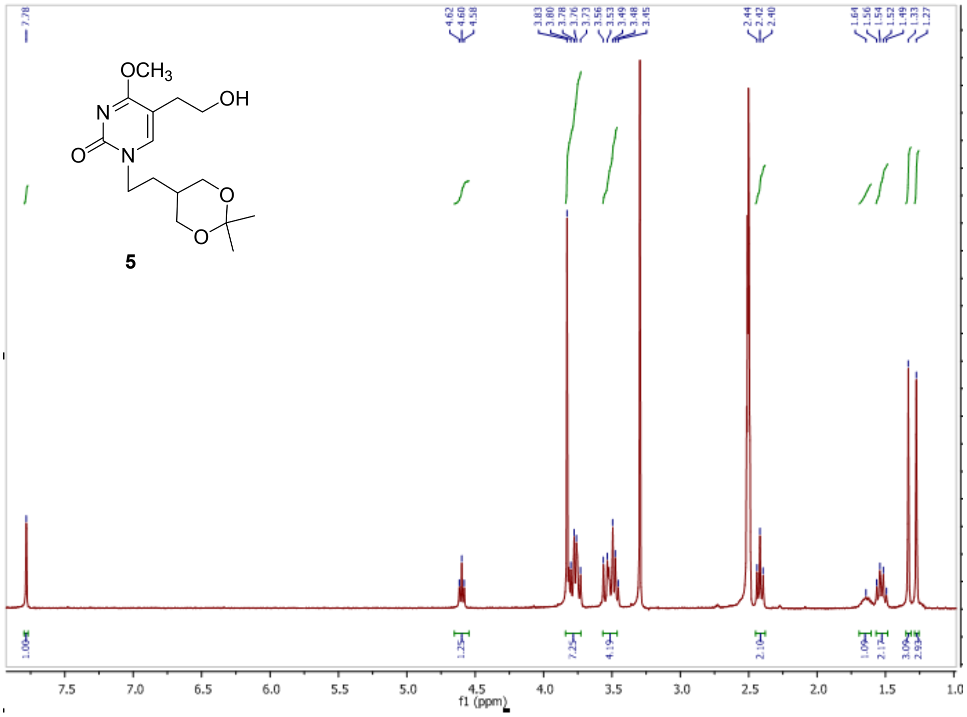

Figure S4. ^{13}C-NMR spectrum of compound **5**.

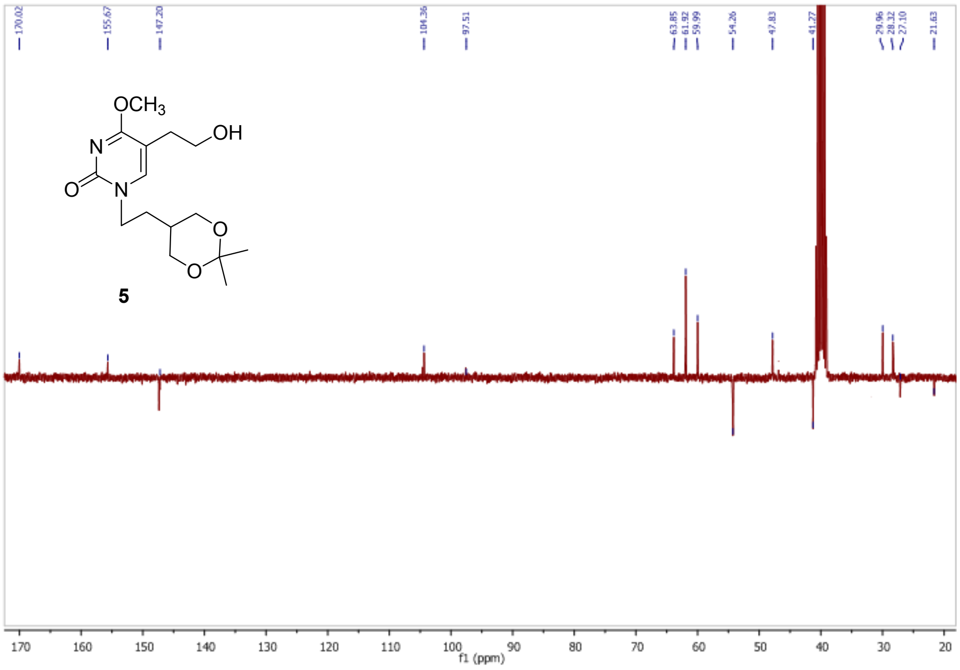

Figure S5. **(a)** ^{1}H-NMR spectrum of compound **7**. **(b)** A part of the ^{1}H-NMR spectrum of **7** displaying H-F coupling in C-5 side chain of the pyrimidine moiety.

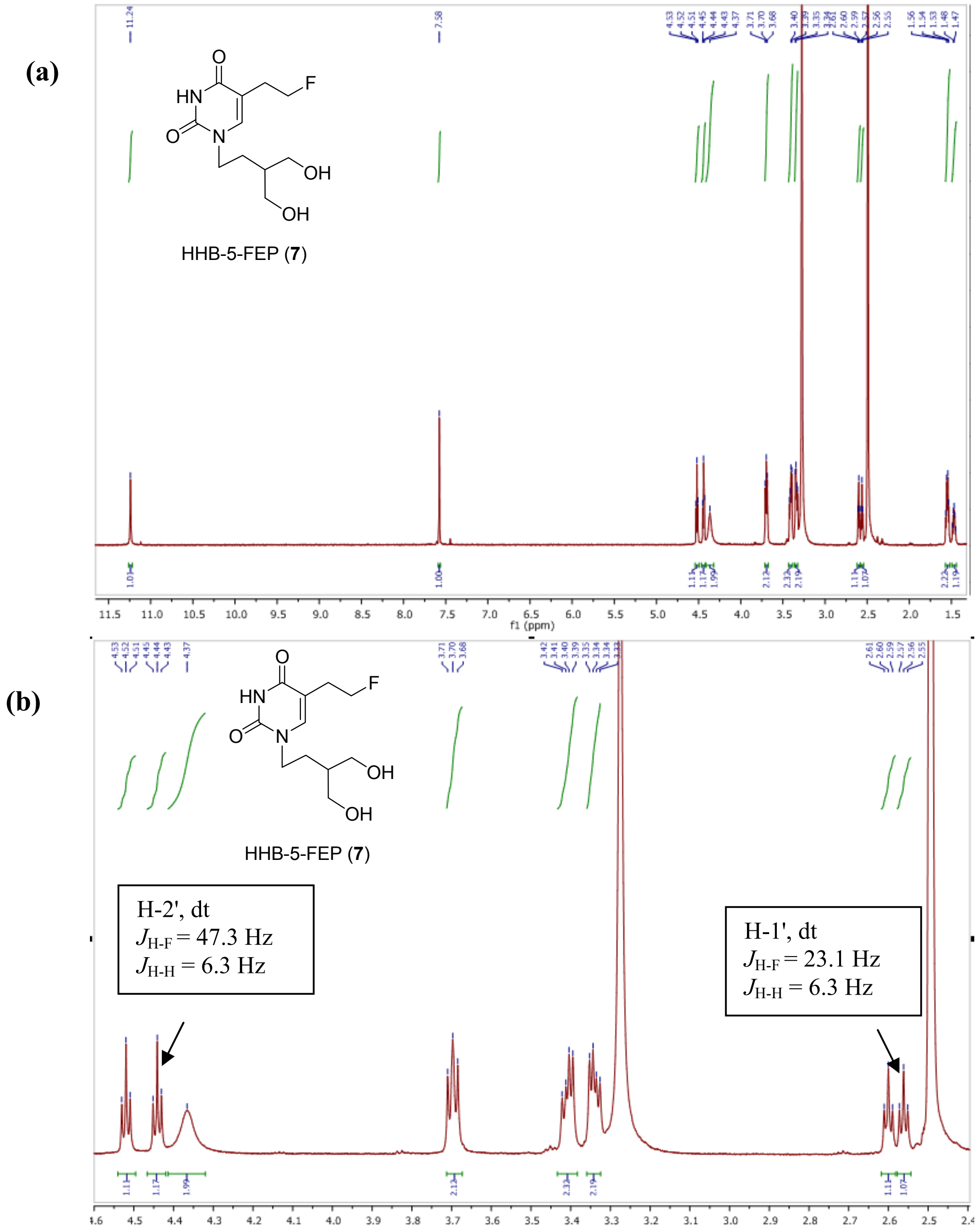

Figure S6. A part of the ^{13}C-NMR spectrum of compound **7** displaying C-F coupling in C-5 side chain of the pyrimidine moiety.

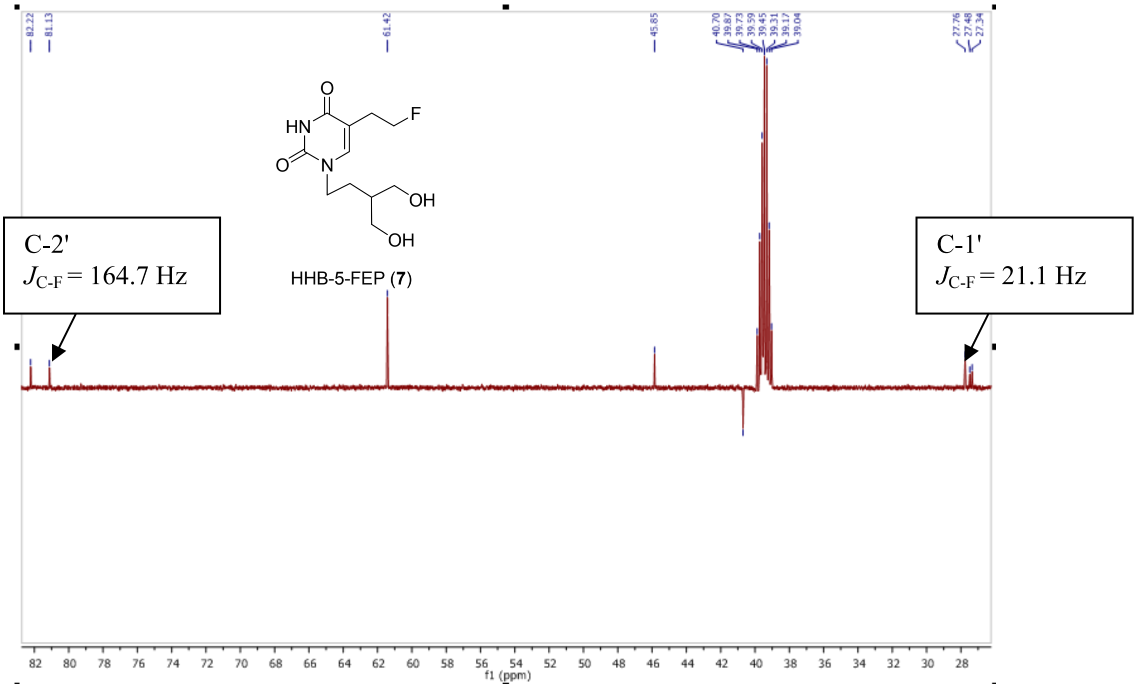

Figure S7. ^{1}H-NMR spectrum of compound **8**.

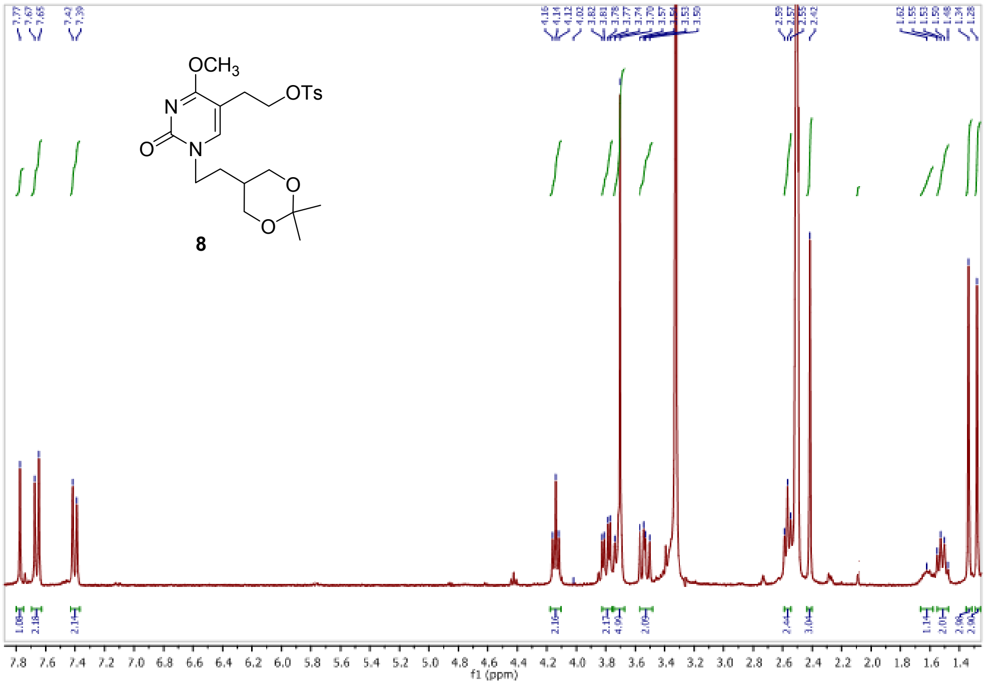

Figure S8. ^{13}C-NMR spectrum of compound **8**.

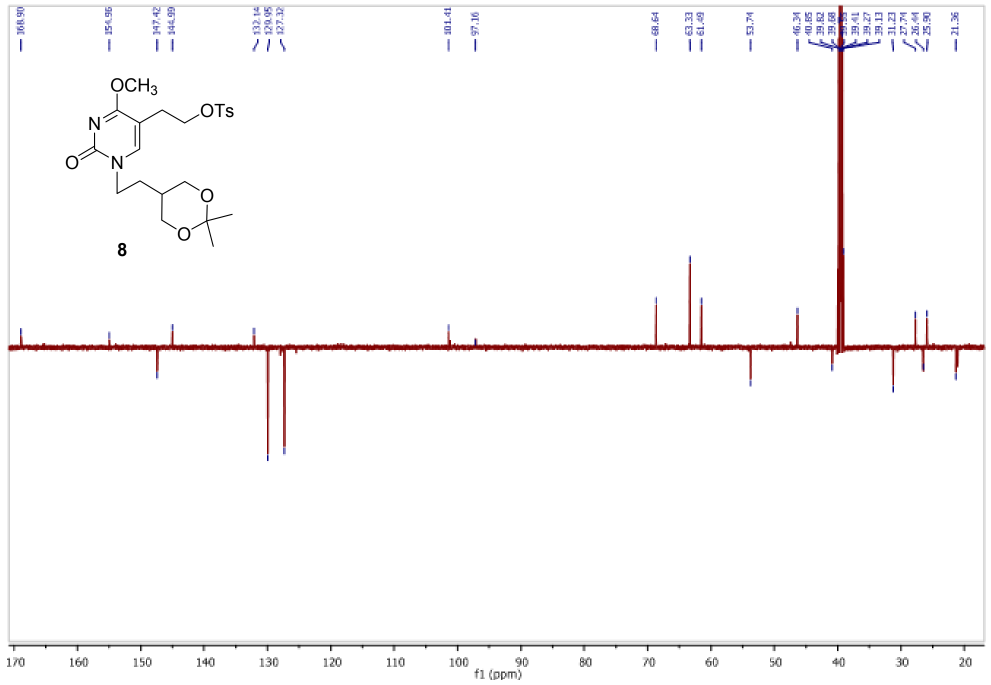

Figure S9. Analytical HPLC chromatogram of [^{18}F]**7** coinjected with the nonradioactive reference compound **7**.

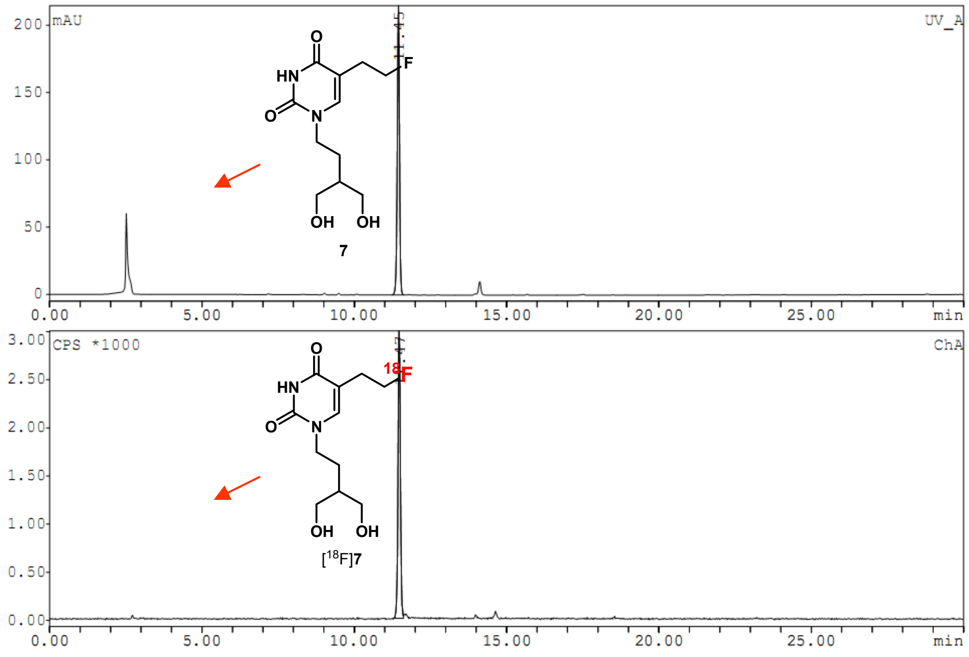

Supplement: Supplementary file 1 [file molecules-18-08535-s001.pdf]
